# Kidney Transplant Outcome Is Associated with Regulatory T Cell Population and Gene Expression Early after Transplantation

**DOI:** 10.1155/2019/7452019

**Published:** 2019-01-08

**Authors:** Magdalena Krajewska, Katarzyna Kościelska-Kasprzak, Dorota Kamińska, Marcelina Żabińska, Marta Myszka-Kozłowska, Agnieszka Gomułkiewicz, Piotr Dzięgiel, Marian Klinger

**Affiliations:** ^1^Department of Nephrology and Transplantation Medicine, Wroclaw Medical University, Borowska 213, 50-556 Wrocław, Poland; ^2^Department of Histology and Embryology, Wroclaw Medical University, Chałubińskiego 6a, 50-368 Wrocław, Poland

## Abstract

Successful long-term kidney allograft survival with parallel reduction of complications resulting from prolonged immunosuppressive treatment is a goal in kidney transplantation. We studied the immune changes in cell phenotypes and gene expression induced by kidney transplantation. Our goal was to find a phenotypic and/or transcriptional pattern that might be considered prognostic for the kidney transplant outcome. The analysis was performed prospectively on 36 KTx recipients sampled during the first year and followed for five years after transplantation and on 40 long-term KTx recipients (7.9 ± 2.2 y. post-KTx). The research involved flow cytometry assessment of lymphocyte subpopulations (including Tregs and CD3^+^CD8^+^CD28^−^ lymphocytes) and gene expression analysis of immune-related genes (*CD4*, *CD8*, *CTLA4*, *GZMB*, *FOXP3*, *IL10*, *IL4*, *ILR2A*, *NOTCH*, *PDCD1*, *PRF1*, *TGFB*, and *TNFA*). The analysis of patterns observed over the first post-KTx year was confronted with control, pretransplant, and long-term transplant results. Treg counts at months one and three post-KTx correlated positively with the current and future allograft function. *FOXP3* gene expression at month one post-KTx was also associated with long-term allograft function. The KTx-induced CD3^+^CD8^+^CD28^−^ population correlated with *GZMB* and *PRF1* expression and suggested their cytotoxic properties. The size of the Treg population and regulatory *FOXP3* gene expression in the early period after transplantation are associated with kidney transplant outcome. The outlined predictive power of the Treg population needs to be investigated further to be confirmed as one of the immune monitoring strategies that may help achieve the best long-term kidney allograft outcomes.

## 1. Introduction

The kidney recipient's immune status and sensitization, quality of organs, and immunosuppressive treatment are some of the factors that determine graft survival and future function of a kidney transplant. Moreover, successful long-term kidney allograft survival may be hindered by a wide range of complications resulting from prolonged immunosuppression as well as suboptimal efficiency of that treatment.

The improvement in long-term allograft survival is still a goal in kidney transplantation with induction of donor-specific tolerance being an ideal target. Data found in the literature point to some cellular and transcriptional signatures of operational tolerance in kidney transplantation [1, 2]. On the other hand, it was shown that only 3.5% of stable kidney allograft recipients exhibited a gene expression profile of operational tolerance, a frequency much lower than that observed in liver transplant recipients [3].

A lot of experimental as well as clinical research performed in the recent years has focused on regulatory T cells (Tregs) and their balance with effector cells to identify the bases of immune tolerance. Regulatory T cells, a subset of T cells expressing CD4, CD25, and the transcription factor Foxp3, are a highly suppressive population constituting approximately 5% to 10% of CD4^+^ T cells that has potent immune regulatory characteristics [4–6]. It is accepted that at the time and shortly after transplantation, Tregs help to prevent initial priming of memory alloreactive T cell response and are involved in induction of allograft tolerance. Graft-protective Tregs are derived *in vivo* from naturally occurring FoxP3^+^ CD4^+^ Tregs (nTregs) and are also generated in the periphery from nonregulatory FoxP3^−^ CD4^+^ cells (iTregs) [5].

The major hallmark of Tregs is the forkhead box P3 (FoxP3) transcription factor whose expression and activity are regulated by multiple factors, including Helios and SATB1 [7]. Also, the suppressive function of Tregs correlates with the methylation status of the Treg-specific demethylated region (TSDR) within the FoxP3 gene locus. The demethylation of this region regulates FoxP3 gene transcription, transforms non-Treg cells to Tregs, and maintains the Treg suppressive function [8].

The observed Treg activity modulated via the FoxP3 transcription factor is dependent on expression of a complex array of proteins such as costimulatory cytotoxic T cell antigen 4 (CTLA4) or glucocorticoid-induced TNFR family-related protein (GITR) [9]. Treg-suppressive activity is mediated by many factors, *inter alia* by secretion of immunosuppressive cytokines (interleukin 10, TGF-*β*) or alteration of dendritic cells activating capacity and cytolysis (delivery of granzymes and perforins) [10, 11].

When isolating Tregs for clinical cell therapy, FoxP3 intracellular staining is omitted and replaced by CD127, the *α*-chain of the IL-17 receptor, which inversely correlates with FoxP3 expression. Low CD127 expression can be used in conjunction with CD4 and CD25 to isolate a highly suppressive Treg population [12]. The freshly isolated CD4+CD25+CD127− T cells were shown to be homogeneously demethylated at their TSDR [13]. According to the Barcelona Consensus on Biomarker-Based Therapeutic Drug Monitoring in Solid Organ Transplantation [14], the phenotype that best characterizes Tregs is defined as CD4^+^CD25^high^FoxP3^+^CD27^+^CD127^low/-^ with the simplified CD3^+^CD4^+^CD25^high^CD127^low/-^ staining accepted for detection of Tregs [12, 15].

Chronic antigenic stimulation induces gradual decrease in CD28 expression on the surface of CD8^+^ T cells that results in expansion of highly antigen-experienced CD8^+^CD28^−^ T cell populations [16, 17]. Although CD28 loss was shown to be related to normal aging, it is enhanced in clinical settings related to chronic antigen exposure including autoimmune diseases [18] and transplantation. It was suggested that a subset of CD8^+^CD28^−^ T cells could be immunosuppressive and promote tolerance in transplant recipients. However, it is also accepted that this population is phenotypically variable with both immunoregulatory and cytotoxic properties [19].

The present research was designed to investigate the immune changes induced by kidney transplantation (KTx). We analyzed the immune cell phenotypes, including Tregs and CD3^+^CD8^+^CD28^−^ cells in relation to a number of immunomodulatory and cytotoxic gene expressions in a group of kidney transplant recipients. Our goal was to find a simple phenotypic and/or transcriptional pattern that might be considered prognostic of the kidney transplant outcome. The analysis of patterns observed over the first post-KTx year was confronted with control, pretransplant, and long-term transplant results.

## 2. Material and Methods

### 2.1. Material

The study was approved by the Bioethical Committee of Wroclaw Medical University and performed in accordance with the World Medical Association Declaration of Helsinki. The participants, who were the patients of the transplant or dialysis units of the Department of Nephrology and Transplantation Medicine, Wroclaw Medical University (Wroclaw, Poland), were included in the study after giving their informed consent. The transplantation procedure was performed solely for medical reasons and was not a part of the present study. All of the recipients received kidney allografts from standard criteria deceased donors (mean kidney donor risk index, KDRI: 1.05 ± 0.26) in the Polish national transplant program coordinated by the Polish Transplant Coordinating Center Poltransplant for end-stage renal disease treatment.

The analysis was performed prospectively on 36 KTx recipients, who survived at least a year after KTx and were then followed for five years after KTx. Analysis also included 40 long-term kidney transplant recipients. All patients were treated with renal-replacement therapy prior to transplantation and received their first transplant with a negative cytotoxic crossmatch. Detailed characteristics of transplant study groups are provided in Table 1. Induction therapy with basiliximab was used in two prospective KTx recipients. All acute rejection episodes were biopsy proven to be T cell-mediated and were treated with boluses of methylprednisolone. No plasmapheresis, IVIG, or ATG was administered. Prospective transplant patients were analyzed over the first year after KTx and the long-term group 7.9 ± 2.2 years after KTx. One patient from the prospective group lost the allograft within the first year post-KTx due to active thrombotic microangiopathy.

In the prospective group the blood samples were collected at five time points: during the first week post-KTx, around month one, month three, month six, and month 12 post-KTx. Kidney function was presented as estimated glomerular filtration rate (eGFR) and calculated using the Modification of Diet in Renal Disease (MDRD) study formula.

18 healthy volunteers (10 males, 8 females; age 46 ± 11 y.) served as the control group. Also, 21 hemodialysis patients from the kidney transplant waiting list (8 males, 13 females; age 46 ± 11 y.; time on dialysis 51.9 ± 46.5 y.) were included in the cell phenotyping study.

### 2.2. Gene Expression

The research involved peripheral blood mononuclear cell (PBMC) gene expression analysis of *CD4*, *CD8*, *CTLA4*, *GZMB*, *FOXP3*, *IL10*, *IL4*, *ILR2A*, *NOTCH*, *PDCD1*, *PRF1*, *TNFRSF18, TGFB*, and *TNFA* genes, referenced to 18S rRNA. 2 × 10^6^ PBMCs were isolated from heparinized blood using density gradient centrifugation on Histopaque 1.077 (Sigma) and washed with PBS. The RNA was purified with RNA Blood Mini Kit (Qiagen) including genomic DNA removal with RNase-free DNase (Qiagen), according to the manufacturer's protocol. The samples were reversely transcribed with a High-Capacity cDNA Reverse Transcription Kit (Applied Biosystems). 10 *μ*l of final reaction volume in 100 *μ*l of TaqMan PCR Master Mix was applied to each channel of a custom-designed low-density array (TaqMan) and analyzed on a TaqMan 7900HT instrument. The results are presented as ΔCT = CT_gene_ − CT_18S_ or as ΔΔCT = ΔCT_mean control sample_ − ΔCT_test sample_. The mean control sample was the mean value of ΔCTs in the control group.

### 2.3. Cell Phenotypes

#### 2.3.1. Antibodies

The following mouse anti-human antibodies (Becton Dickinson) were used for cell phenotyping: anti-CD3-APC (clone UCHT1), anti-CD4-PerCP (clone SK3), anti-CD25-FITC (clone M-A251), anti-CD28-PE (clone CD28.2), anti-CD8-FITC (RPA-T8), anti-CD127-PE (clone hIL-7R-M21), and the four-color BD Multitest (CD3/CD16+CD56/CD45/CD19).

#### 2.3.2. Whole Blood Staining

**Figure 1:**
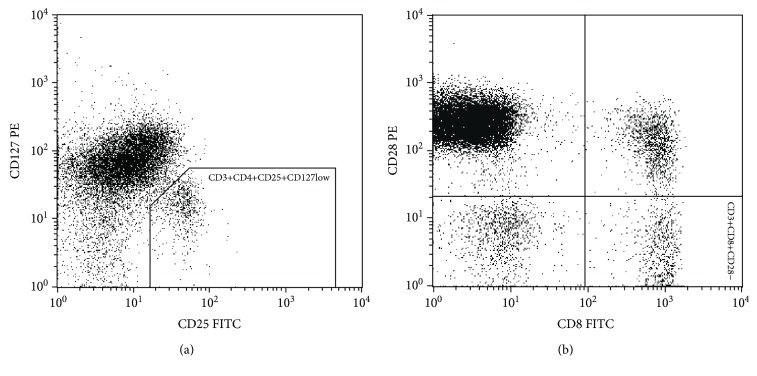
Flow cytometry gating strategies. The lymphocyte population was selected based on FSC/SSC scatters: (a) CD127 vs. CD25 dot plot of CD4+ CD3+ lymphocytes; (b) CD28 vs. CD8 dot plot of CD3+ lymphocytes.

The samples of EDTA-anticoagulated blood were stained with anti-CD3-APC, anti-CD4-PerCP, anti-CD25-FITC, and anti-CD127-PE for CD3^+^CD4^+^CD25^+^CD127^low^ phenotyping and with anti-CD3-APC, anti-CD8-FITC, and anti-CD28-PE for CD3^+^CD8^+^CD28^−^. After incubation the samples were lysed with BD FACS Lysing Solution, washed with PBS, and subjected to analysis on a FACSCalibur flow cytometer (Becton Dickinson). The gating strategy is shown in Figure 1. The absolute number of T cells per *μ*l of blood was determined with the BD Multitest in Trucount tubes (Becton Dickinson) according to the manufacturer's protocol. The subpopulations were measured in relation to T cells, which also enabled their enumeration per *μ*l of blood.

### 2.4. Statistical Methods

Statistical analysis was performed with the STATISTICA v.13 statistical package (StatSoft, Poland). The numerical variables were tested for outliers and normality and presented as mean ± standard deviation or median + interquartile range. As mostly the normality assumption was not fulfilled, the Mann-Whitney *U*-test, Wilcoxon paired test, Friedman ANOVA, and Spearman correlation were used. The significance level of *α* = 0.05 and the Bonferroni-Holm correction for multiple testing [20] were included for cell populations and expression of data families (the adjusted *p* values are shown).

## 3. Results

The blood samples for the study were obtained from the prospectively analyzed KTx recipients at 4 ± 2 days post-KTx (eGFR median 21, IQR 9–33 ml/min/1.73 m^2^), 37 ± 8 days post-KTx (eGFR median 41, IQR 32–54 ml/min/1.73 m^2^), 108 ± 26 days post-KTx (eGFR median 42, IQR 38–56 ml/min/1.73 m^2^), 218 ± 59 days post-KTx (eGFR median 49, IQR 41–59 ml/min/1.73 m^2^), and 421 ± 63 days post-KTx (eGFR median 52, IQR 43–62 ml/min/1.73 m^2^). During the study period, one graft loss was observed (three months after KTx, due to graft thrombosis experienced by a recipient with complement cascade mutation).

The study material also included long-term kidney transplant recipients who were age (at the time of sampling) and gender matched. Moreover, the long-term kidney transplant recipients presented eGFR one year after transplantation similar to that of prospectively analyzed recipients (median 51, IQR 40–63 vs. 46, 41–58; *p* = 0.680).

No relationship between recipient's age and transplant outcome was observed. Both donor age and KDRI were negatively associated with subsequent allograft function (Table 2), and the correlation was still observed at month 60 post-KTx (*r*_s_ = −0.47, *p* = 0.009, and *r*_s_ = −0.44, *p* = 0.015, respectively). There was also no relationship between donor gender, cold or warm ischemia time, and transplant outcome.

### 3.1. Cell Phenotypes

In the current study we measured the absolute cell counts of T, CD4^+^, and CD8^+^ T, B (CD19+), and NK cells (CD16+/CD56+) together with Tregs (CD3^+^CD4^+^CD25^+^CD127^low^ phenotyping) and CD3^+^CD8^+^CD28^−^ cells.

Treg absolute count was not related to age in healthy controls and dialysis patients. However, following KTx, the negative correlation between the recipient age and Treg population was observed (m. 1 *r*_s_ = −0.36, *p* = 0.036; m. 3 *r*_s_ = −0.54, *p* < 0.001; m. 6 *r*_s_ = −0.42, *p* = 0.014; and m. 12 *r*_s_ = −0.37, *p* = 0.041). Similarly, recipient age was negatively associated with total T lymphocytes, CD4+ T, and NK cells, which were not influenced by age in dialysis and healthy control groups. CD8+ T lymphocytes were negatively related to age in healthy controls (*r*_s_ = −0.50, *p* = 0.022), but this relation was not observed in dialysis patients and transplant recipients. The gender was not related to cell counts in any of the groups.

The absolute counts of the T and T CD4^+^ lymphocyte populations were similar in the dialysis and control groups (Table 3). The dialysis patients presented significantly lower T CD8^+^ (median 325, IQR 364–397 vs. 399, 336–512; *p* = 0.047), B (median 109, IQR 88–215 vs. 230, 145–289; *p* = 0.012), and NK (median 100, IQR 55–120 vs. 273, 175–305; *p* < 0.001) counts.

The cell absolute counts during week one after KTx showed a decrease related to blood loss after transplant surgery but also initiation of immunosuppressive therapy. After one month, the observed T and T CD4^+^ cell counts were normalized. There was a large drop in T CD8^+^ counts induced by the KTx procedure, and it was reversed over the first six months. The NK cell population gradually increased after KTx to reach control values after six months.

After the initial fluctuations in cell counts, the transplant population generally presented lymphocyte counts similar to the control group, except for B cells, which dropped from normal values of median 223, IQR 153–315/*μ*l (observed at month one post-KTx) to median 111, and IQR 59–158/*μ*l over the first year (*p* < 0.001).

The Treg population did not differ between the dialysis group and healthy controls (Table 4). After the absolute count of Tregs was restored following the initial transplantation-related dropdown, it decreased slowly after the first month, and six months after KTx, it was significantly lower than the initial (dialysis) value and the control one (median 40.8, IQR 27.4–53.1 cells/*μ*l blood, *p* = 0.033/0.036). After KTx the ratios of Tregs to total T cells and to CD4^+^ T cells decreased gradually and stabilized after the sixth month, being significantly lower than those of dialysis patients and controls (median 2.6, IQR 2.2–3.3, *p* < 0.001, and median 4.7, IQR 4.1–5.8, *p* < 0.001, respectively).

Dialysis patients presented a significantly greater population of CD3^+^CD8^+^CD28^−^ cells (Table 5) compared to healthy controls both in terms of absolute count (median 86, IQR 14–85.4 vs. 43.1, 14.0–85.4 cells/*μ*l blood, *p* = 0.048) and ratio to total T cells (median 9.7, IQR 5.6–15.5 vs. 3.2, 1.2–6.0%, *p* = 0.004). Shortly after KTx this lymphocyte subpopulation was decreased, then restored over the first three months, and further increased reaching median 216, IQR 89–393 cells/*μ*l blood, 14.7, 7.1–22.6% of T cells and 59.3, 42.3–73.7% of CD8^+^ T cells after one year. In the long-term group, the size of this population is reduced but still significantly higher than for healthy controls.

### 3.2. Gene Expression

We studied the expression of 14 immune-related genes, which could be assigned into an immunomodulatory and protolerogenic pattern (*CD4*, *CTLA4*, *FOXP3*, *IL10*, *PDCD1*, *TGFB*, and *TNFRSF18*) or allograft rejection molecular pattern (*CD8*, *GZMB*, *IL4*, *ILR2A*, *NOTCH*, *PRF1*, and *TNFA*). No association between gene expression and recipient gender or immunosuppression used was observed. There was a positive correlation between *GZMB* expression and recipient age at month one post-KTx (*r*_s_ = 0.48, *p* = 0.003) that was not observed in the control group. The relation, weakly sustained also at month three (*r*_s_ = 0.36, *p* = 0.037), was not present later after transplantation.

Gene expression analysis showed that transplant recipients presented an increased expression of immunomodulatory genes (weakly affected by the time from KTx) compared to healthy controls (Table 6):

- *CD4* with a 1.9-fold mean increase over the first post-KTx year was constantly elevated from 1.7- to 2.1-folds, with no significant changes throughout the whole year
- *IL10* increased greatly during the first week (18.5-folds, *p* < 0.001), and afterwards, it was elevated not more than 5-folds over the first six months post-KTx (*p* < 0.001)
- *TGFB* was higher by not less than 1.9 times (2.1-fold mean increase over the first post-KTx year)
- *TNFRSF18* (1.9-fold mean increase over the first post-KTx year)

On the other hand, the expression of rejection-related molecules (CD8 and granzyme B) immediately post-KTx was similar to that of healthy controls and increased with time. CD8 expression was significantly higher compared to controls at month three, six, and 12 (*p* < 0.001). The level of expression increased up to four times, reaching the maximum at month six (*p* < 0.001). *GZMB* expression was the highest at month six (3.9-folds compared to healthy controls). The expression rose from week one to month six (*p* < 0.001), followed by a slight decrease. Long-term recipients' *GZMB* expression did not differ from that of healthy controls.

### 3.3. Correlations of Cell Subpopulations and Gene Expression with Clinical Outcome

#### 3.3.1. Acute Rejection and Delayed Graft Function

When patients experienced an acute rejection (AR) during the first six months post-KTx, month one samples presented a decreased population of CD3^+^CD8^+^CD28^−^ cells (median 16.3, IQR 5.8–39.5 vs. 48.9, 19.9–102 cells/*μ*l blood, *p* = 0.011), accompanied by decreased total T cells (median 888, IQR 569–1387 vs. 1503, 1002–1855 cells/*μ*l blood, *p* = 0.024), CD8^+^ T cells (median 70, IQR 48–146 vs. 163, 102–336 cells/*μ*l blood, *p* = 0.038), and NK cells (median 45, IQR 31–162 vs. 151, 99–229 cells/*μ*l blood, *p* = 0.037), compared to AR-free recipients. AR episodes were not associated with any gene expression. Moreover, delayed graft function did not influence the studied lymphocyte subpopulations or gene expression.

#### 3.3.2. Kidney Allograft Function

The Treg number per *μ*l of blood observed at one and three months after KTx was associated with the transplant outcome. Treg count at month one post-KTx correlated positively with allograft function (Table 2). Also, the absolute Treg count at month three correlated positively with the long-term allograft function. The ratio of Tregs to total T cells three months after transplantation correlated with eGFR observed six months post-KTx (*r*_s_ = 0.48, *p* = 0.006), as well as 12 months (*r*_s_ = 0.50, *p* = 0.004). Treg count observed after the third month was not associated with kidney function. The other cell populations, including CD3^+^CD8^+^CD28^−^ cells, were not related to allograft function.

Two of the immunomodulatory genes were also associated with kidney function (Table 2). *FOXP3* month one gene expression positively correlated with graft function at month three (*r*_s_ = 0.36, *p* = 0.030), month six (*r*_s_ = 0.46, *p* = 0.007), month 12 (*r*_s_ = 0.45, *p* = 0.009), and month 24 (*r*_s_ = 0.37, *p* = 0.043). Moreover, *FOXP3* month three expression correlated positively with month 12 eGFR (*r*_s_ = 0.37, *p* = 0.040), month 24 (*r*_s_ = 0.39, *p* = 0.034) and year five (*r*_s_ = 0.45, *p* = 0.014). *IL10* month one expression correlated positively with long-term graft function (eGFR m. 24 *r*_s_ = 0.50, *p* = 0.005; y. 5 *r*_s_ = 0.55, *p* = 0.002).

The positive influence of Tregs on allograft survival counterbalanced the negative impact of donor age on the allograft outcome.

### 3.4. Cell Subsets and Gene Expression

Gene expression analysis was performed in total PBMCs, and no data is available for gene expression in individual T cell subpopulations. However, some associations between gene expression levels and the percentage of Tregs or the CD3+CD8+CD28− population were observed.

First of all, we noted the expected correlation between *FOXP3* gene expression and the size of the Treg population, in particular the ratio of Tregs to total T cells (m. 1 *r*_s_ = 0.46, *p* = 0.006; m. 3 *r*_s_ = 0.43, *p* = 0.009). On the other hand, we observed the associations between the *CD8* gene expression and the CD3+CD8+CD28− ratio to T cells (m. 1 *r*_s_ = 0.54, *p* < 0.001; m. 3 *r*_s_ = 0.40, *p* = 0.016; and m. 12 *r*_s_ = 0.52, *p* = 0.004). CD3+CD8+CD28− cell percentage was also associated with expression of rejection-associated molecules, like granzyme B or perforin 1 (*GZMB*: m. 1 *r*_s_ = 0.42, *p* = 0.011; *PRF1*: m. 1 *r*_s_ = 0.37, *p* = 0.027; m. 3 *r*_s_ = 0.45, *p* = 0.007).

## 4. Discussion

We analyzed immune cell populations before and during the first year after kidney transplantation, as well as in long-term stable allograft recipients together with the immune cell-related gene expression.

We observed that end-stage renal disease and dialysis resulted in a decrease of CD8^+^ T, B, and NK cells in contrast to T and CD4^+^ T lymphocyte counts resembling that of healthy volunteers. Kidney transplantation did not generally influence the total T and CD4^+^ T lymphocytes; however, it resulted in gradual expansion of NK cells. As a result of transplantation and introduction of immunosuppression, we observed an immediate excessive decrease of the CD8^+^ T lymphocyte population, which was restored after six months, while the B cell population presented a slow decrease over the first six months post-KTx.

Gene expression analysis showed that transplant recipients presented an increased expression of immunomodulatory genes: *CD4*, *IL10*, *TGFB*, and *TNFRSF18*, compared to healthy controls. On the other hand, the expression of rejection-related molecules: CD8 and granzyme B, immediately post-KTx was similar to healthy controls and increased with time.

### 4.1. Role of Tregs in Kidney Transplantation

In our study we paid special attention to Treg cells and we found that regulatory T cells slightly and gradually decreased after KTx, and in the early posttransplant phase, their count was associated with subsequent allograft function up to one year after transplantation. Also, two of the Treg-related immunomodulatory genes, *FOXP3* and *IL10*, were associated with a subsequent kidney function.

In stable kidney transplant recipients, the number of CD25^high^CD4^+^ T cells in the peripheral blood did not differ from healthy controls, whereas it was significantly lower in the recipients with biopsy-proven chronic rejection of the allograft [21]. Stable kidney recipients also presented strong coexpression of IFN-*γ* and FoxP3 by CD4+CD25+ cells [22]. There is increasing evidence of immunosuppression mediated by IFN-*γ*-producing Tregs. IFN-*γ*+Tregs are induced by IFN-*γ* and interleukin 12 and represent initial response to immune stimulus [10].

In our study we observed that the Treg population slowly decreased after KTx with significantly lower values than controls and dialysis patients from month six posttransplantation. San Segundo et al. [23] observed low levels of Tregs after KTx for six months after transplantation and their recovery to almost basal levels during the first posttransplant year, which may be explained by the high burden of immunosuppression in the early posttransplant period. On the contrary, we previously showed that stable kidney transplant recipients chronically presented lower counts of Tregs compared to controls and stable hand transplant recipients despite their higher immunosuppression load [24]. Moreover, long-term treatment with calcineurin inhibitors in contrast to mTOR inhibitors in stable human renal transplantation was linked to decreased Treg counts [25].

Although some authors observed a link between Treg numbers before [26] and after transplantation and rejection incidence [27, 28], none of them were able to demonstrate any predictive value of circulating Tregs for rejection or graft survival. Tregs examined in graft and urine present a stronger link to transplant outcomes [29]. Also, in our study we did not find any association between circulating Treg numbers and occurrence of rejection episodes. The paper recently published by Ma et al. showed lower counts of CD4^+^CD25^+^FoxP3^+^ T cells in rejecting graft recipients compared to stable patients [30]. On the other hand, the presence of increased pretransplantation counts of activated Tregs with the phenotype CD4^+^CD25^high^CD62L^+^CD45RO^+^ was associated with increased risk of acute rejection [28].

Despite the lack of Treg relations to rejection episodes, we were able to demonstrate that the Treg absolute number in peripheral blood observed at months one and three after kidney transplantation was associated with the one-year transplant outcome. Similarly, it was also reported that high Treg counts at both months six and 12 after kidney transplantation correlated with better long-term graft survival [31] and chronic rejection was associated with a decreased number of CD4^+^CD25^high^FOXP3^+^ T cells [32].

In our research we noted the correlation between Treg number and *FOXP3* gene expression early after transplantation (up to month three). Also, *FOXP3* expression at months one and three correlated with improved long-term allograft function. The published reports about the prognostic value of *FOXP3* expression are conflicting with the expression linked to both good and bad prognosis and mostly regarding its intragraft expression [33]. *FOXP3* gene expression in peripheral blood early after transplantation was significantly higher in rejection-free patients as compared to the rejection group with the highest differences during the first three months [34]. Renal transplant recipients with chronic rejection presented a decreased number of peripheral CD4^+^CD25^+^FoxP3^+^ Tregs compared to those with stable renal graft function [2, 21, 35]. Additionally, the level of *FOXP3* transcripts was lower in recipients with chronic rejection [36]. However, Ashton-Chess et al. [37] reported that peripheral blood *FOXP3* levels could not distinguish between rejection and nonrejection status in contrast to granzyme B that decreased in patients with chronic antibody-mediated rejection. The report by Iwase et al. [38] showed significantly lower levels of FoxP3 and granzyme B in peripheral blood during chronic rejection.

### 4.2. Role of CD28^−^ Cells in Kidney Transplantation

We also focused on CD3^+^CD8^+^CD28^−^ cells which are considered a nonhomogenous population that is believed to include the immunomodulatory subset of cells but also annotated to cytotoxicity. Our results showed that the population was higher in dialysis patients compared to healthy controls and grew further after kidney transplantation. Gene expression results showed some associations between rejection-associated molecules like granzyme B or perforin 1 and the CD3^+^CD8^+^CD28^−^ population. Despite clear expansion of that lymphocyte subset that is likely to be due to chronic antigenic stimulation, we did not observe any association with allograft function.

CD28 antigen is essential for the activation of T cells (both naïve and antigen-experienced cells), providing a second (costimulatory) signal which is essential for their full activation [39]. Many human disorders are associated with an increase of the peripheral blood CD3^+^CD8^+^CD28^−^ population [40]. Blockade of the second activation signal involving CD28 is one of the most promising methods of immunosuppression. However, costimulation blockade-resistant rejection is described in literature [41, 42].

After a certain number of cell divisions, human lymphocytes become immunosenescent, terminally differentiated, with a lost expression of CD28 but with sustained ability to proliferate [19, 43]. Chronic inflammation and some viral infections after transplantation (esp. CMV) are related to expansion of CD28-negative T cells [44].

After transplantation, chronic alloantigenic stimulation may result in gradual accumulation of late-differentiated T cells characterized by CD28 loss [16] of a phenotype related to allograft rejection [42]. We observed that the CD3^+^CD8^+^CD28^−^ population is already expanded in dialysis patients, and it gradually increases with time after transplantation.

Proliferating CD28^−^ memory, CD8^+^ T cells produce high amounts of IFN-*γ* and tumor necrosis factor *α* [19]. Experimental data showed that isolated CD28^+^ T cells obtained from end-stage renal disease patients exposed to allostimulation in the presence of belatacept differentiated into CD28^−^ T cells producing high amounts of IFN-*γ* and were able to mediate allogeneic response [45]. Higher percentages of CD8^+^CD28^−^ T cells were observed in long-term kidney allograft recipients with chronic rejection when compared either to stable allograft recipients or to healthy controls [46]. In stable long-term kidney transplant recipients, CD28-negative T cells have been recognized among other CD8-positive cells that correlate with allograft dysfunction [47]. Our study showed that the CD3^+^CD8^+^CD28^−^ cell count correlated positively with the observed *CD8*, *GZMB*, and *PRF1* expression, which may imply their cytotoxic properties.

On the other hand, as CD8^+^CD28^−^ T cells are considered a heterogeneous population, it was suggested that their accumulation due to long-lasting antigen stimulation after organ transplantation may also exert immunosuppressive activity [16] and lead to operational tolerance [48] with stabilization of the allograft function. In liver recipients from living donors, higher counts of CD8^+^CD28^−^ T cells correlated with improved graft function and reduction of acute rejection episodes [49]. After induction therapy of kidney transplant recipients with alemtuzumab, CD8^+^CD28^−^ T cells recovered significantly faster than CD4 T cells. Additional *in vitro* experiments revealed the potential of CD8^+^CD28^−^ T cells to suppress proliferation of CD4+ T cells. Moreover, acute rejection episodes occurred in patients with the lowest count of CD8^+^CD28^−^ cells [50]. That is in agreement with our observation that the month one absolute count of CD3^+^CD8^+^CD28^−^ cells was significantly lower in the acute rejection-positive group compared to rejection-free recipients. The observed expansion of CD3^+^CD8^+^CD28^−^ with time after transplantation may result from aging of the recipients or senescence of the recipient's immune system due to exposition to alloantigens, viral infection, or alterations of the internal environment.

### 4.3. Limitation of the Study

We were not able to obtain the pretransplant blood samples from KTx recipients. To better describe the transplant-induced immune changes, we included data obtained for hemodialysis patients from the kidney transplant waiting list, which could be considered an estimation of the pretransplant immune state. Gene expression analysis was performed in total PBMCs, and no data is available for gene expression in individual T cell subpopulations.

## 5. Conclusion

The general immune status after kidney transplantation is characterized by expansion of CD8+ T cells, restoration of NK cells, and limitation of B lymphocytes. Allograft function is associated with the early posttransplant size of the Treg population and *FOXP3* gene expression. Treg population presents a time-dependent reduction after kidney transplantation, which may be a result of immunosuppressive treatment. Chronic stimulation with kidney-related donor antigens leads to the ongoing expansion of the CD8+CD28− T cell population with suggested cytotoxic properties.

The Treg count assessed by a simple extracellular staining was shown to be related to kidney transplant outcome. The outlined predictive power of this marker needs to be investigated further to be confirmed as one of the immune-monitoring strategies that may help achieve the best long-term kidney allograft outcomes.

## Figures and Tables

**Table 1:** Clinical characteristics of the transplant study groups.

	Short term (prospective)	Long term (retrospective)	*p* value
Recipient gender	25 M/11 F	27 M/13 F	0.527

Age at KTx	52.9 ± 11.2 y (56; 46.5–62 y)	41.0 ± 13.5 y (42; 27.5–52.5 y)	<0.001

Age during the study	52.9 ± 11.2 y (56; 46.5–62 y)	48.9 ± 13.2 y (52; 37–59)	0.153

Time from KTx to examination	1°: 3–5 days	7.9 ± 2.2 y (7; 6–10 y)	<0.001
	2°: 30–42 days		
	3°: 90–123 days		
	4°: 195–225 days		
	5°: 365–443 days		

RRT before tx	24.7 ± 13.6 y (24; 14–33 y)	23.5 ± 17.2 y (19; 12–29 y)	0.398

Donor gender	26 M/10 F	27 M/13 F	0.423

Donor age	45.6 ± 14.2 y (53; 33–56.5 y)	43.5 ± 13.2 y (45; 37–52 y)	0.243

HLA mismatches	From 1 to 6, median 4	From 1 to 5, median 3	

Panel-reactive antibodies (PRA)	16 recipients (3–61%)	19 recipients (4–50%)	

Cold ischemia time	24.2 ± 6.8 h (24; 20–29 h)	25.4 ± 8.2 h (24; 21–29 h)	0.820

Delayed graft function	5 recipients	6 recipients	0.597

Acute rejection episodes	11	15	0.347

Maintenance IS	Tac/MMF/steroids 20	Tac/MMF/steroids 13	
	Tac/Aza/steroids 2	Tac/Aza/steroids 1	
	CsA/MMF/steroids 13	CsA/MMF/steroids 11	
	CsA/Aza/steroids 1	CsA/Aza/steroids 15	

1-year eGFR	51.4 ± 14.1 y (49; 42–60 y)	52.6 ± 16.6 y (51, 40–60 y)	0.844

Abbreviations: IS: immunosuppression; Tac: tacrolimus; MMF: mycophenolate mofetil or sodium; Aza: azathioprine; CsA: cyclosporine A.

**Table 2:** Factors associated with estimated glomerular filtration rate (correlation coeff, *p* value).

	Estimated glomerular filtration rate after KTx					
	1 m.	3 m.	6 m.	12 m.	24 m.	60 m.
Donor age	**-0.57** (<0.001)	**-0.60** (<0.001)	**-0.49** (0.003)	**-0.44** (0.010)	**-0.48** (0.007)	**-0.47** (0.009)
KDRI	**-0.57** (<0.001)	**-0.60** (<0.001)	**-0.48** (<0.001)	**-0.46** (<0.001)	**-0.42** (<0.001)	**-0.44** (<0.001)
Treg/*μ*l m. 1 post-KTx	**0.54** (<0.001)	0.33 (0.054)	**0.41** (0.021)	**0.45** (0.011)	**0.47** (0.010)	**0.48** (0.011)
Treg/*μ*l m. 3 post-KTx	—	0.11 (0.548)	0.19 (0.294)	0.29 (0.109)	**0.55** (0.002)	**0.42** (0.023)
*FOXP3* m. 1 post-KTx	0.32 (0.064)	**0.36** (0.030)	**0.46** (0.007)	**0.45** (0.009)	**0.37** (0.043)	0.17 (0.367)
*FOXP3* m. 3 post-KTx	—	0.16 (0.359)	0.34 (0.052)	**0.37** (0.040)	**0.39** (0.034)	**0.45** (0.014)
*IL10* m. 1 post-KTx	0.28 (0.102)	0.33 (0.050)	0.33 (0.063)	**0.36** (0.044)	**0.50** (0.005)	**0.55** (0.002)

**Table 3:** The absolute counts of lymphocyte populations in examined groups of recipients in relation to controls and hemodialysed patients.

Cells	Control group	Dialysis group	*p* value dialysis vs. control	Time from KTx	Mean ± SD (median; IQR)	*p* value KTx vs. control	*p* value KTx vs. dialysis
T/*μ*l	1317 ± 357 (1299; 1169–1505)	1126 ± 465 (1154; 894–1372)	0.268	1 w.	679 ± 293 (701; 375–824)	**<0.001**	**<0.001**
				1 m.	1499 ± 1106 (1315; 888–1822)	*0.906*	*0.370*
				3 m.	1399 ± 624 (1385; 957–1615)	*1.000*	*0.428*
				6 m.	1502 ± 725 (1461; 1092–1869)	*0.993*	*0.267*
				12 m.	1550 ± 727 (1476; 1073–1904)	*0.936*	*0.345*

T CD4/*μ*l	811 ± 306 (781; 623–844)	812 ± 386 (799; 657–917)	0.801	1 w.	457 ± 196 (483; 274–575)	**<0.001**	**<0.001**
				1 m.	1037 ± 809 (888; 626–1174)	*1.000*	*1.000*
				3 m.	841 ± 352 (783; 669–1082)	*1.000*	*1.000*
				6 m.	827 ± 392 (801; 580–1019)	*1.000*	*1.000*
				12 m.	847 ± 486 (792; 506–1073)	*0.867*	*0.852*

T CD8/*μ*l	424 ± 150 (399; 336–512)	404 ± 358 (325; 364–397)	**0.047**	1 w.	68 ± 70 (52; 17–86)	**<0.001**	**<0.001**
				1 m.	188 ± 171 (137; 70–252)	**<0.001**	**0.007**
				3 m.	250 ± 244 (158; 81–308)	**<0.001**	**0.040**
				6 m.	374 ± 336 (243; 148–512)	*0.136*	*1.000*
				12 m.	416 ± 320 (327; 215–524)	*0.361*	*0.654*

B/*μ*l	234.3 ± 102.1 (230; 145–289)	155.3 ± 115.9 (109; 88–215)	**0.012**	1 w.	179 ± 119 (144; 75–276)	*0.124*	*1.000*
				1 m.	280 ± 241 (223; 153–315)	*0.912*	*0.140*
				3 m.	170 ± 116 (161; 78–254)	*0.054*	*1.000*
				6 m.	117 ± 84 (99; 58–160)	**<0.001**	*0.813*
				12 m.	125 ± 85 (111; 59–158)	**<0.001**	*0.551*

NK/*μ*l	253.3 ± 82.6 (273; 175–305)	97.4 ± 49.7 (100; 55–120)	**<0.001**	1 w.	86 ± 53 (74; 45–118)	**<0.001**	*0.275*
				1 m.	143 ± 96 (121; 54–218)	**<0.001**	*0.194*
				3 m.	188 ± 147 (151; 68–253)	**0.020**	**0.047**
				6 m.	248 ± 209 (227; 102–298)	*0.156*	**0.003**
				12 m.	241 ± 188 (172; 115–314)	*0.270*	**0.003**

**Table 4:** Regulatory T cell absolute and related counts in examined groups of recipients in relation to controls and hemodialysed patients.

Cells	Control group	Dialysis group	*p* value dialysis vs. control	Time from KTx	Mean ± SD (median; IQR)	*p* value KTx vs. control	*p* value KTx vs. dialysis
Treg*/μ*l	60.8 ± 29.8 (54.5; 41.7–71.8)	60.1 ± 28.5 (57.8; 40.5–90.5)	0.745	1 w.	26.9 ± 13.8 (28.5; 14.8–36.8)	**<0.001**	**<0.001**
				1 m.	53.9 ± 34.6 (47.0; 30.4–70.6)	*0.303*	*0.174*
				3 m.	44.6 ± 19.6 (44.1; 34.7–56.5)	*0.105*	*0.061*
				6 m.	40.8 ± 19.6 (40.8; 27.4–53.1)	**0.033**	**0.036**
				12 m.	41.0 ± 22.7 (38.0; 26.0–48.2)	**0.035**	**0.030**

Treg %T	4.6 ± 1.4 (4.5; 3.7–5.3)	5.4 ± 1.5 (5.3; 4.2–6.7)	0.129	1 w.	4.0 ± 1.2 (4.1; 3.2–4.9)	*0.206*	**0.003**
				1 m.	3.8 ± 1.0 (3.8; 3.1–4.5)	*0.077*	**<0.001**
				3 m.	3.2 ± 0.9 (3.2; 2.6–3.8)	**0.002**	**<0.001**
				6 m.	2.9 ± 1.0 (2.6; 2.2–3.3)	**<0.001**	**<0.001**
				12 m.	2.9 ± 1.4 (2.8; 2.1–3.3)	**<0.001**	**<0.001**
				7.9 ± 2.2 y.	2.9 ± 1.1 (2.5; 2.2–3.5)	**<0.001**	**<0.001**

Treg % T CD4	7.2 ± 1.7 (7.4; 5.6–8.3)	7.9 ± 1.9 (7.7; 6.6–9.3)	0.357	1 w.	6.0 ± 1.9 (6.2; 4.8–7.1)	**0.014**	**0.001**
				1 m.	5.4 ± 1.4 (5.3; 4.7–6.1)	**0.002**	**<0.001**
				3 m.	5.3 ± 1.5 (5.2; 4.4–6.3)	**0.001**	**<0.001**
				6 m.	5.1 ± 1.9 (4.7; 4.1–5.8)	**<0.001**	**<0.001**
				12 m.	5.5 ± 2.2 (5.3; 4.2–6.3)	**0.003**	**<0.001**
				7.9 ± 2.2 y.	4.7 ± 1.4 (4.6; 3.5–5.9)	**<0.001**	**<0.001**

**Table 5:** CD8^+^CD28^−^ T cell absolute and related counts in examined groups of recipients in relation to controls and hemodialysed patients.

Cells	Control group	Dialysis group	*p* value dialysis vs. control	Time from KTx	Mean ± SD (median; IQR)	*p* value KTx vs. control	*p* value KTx vs. dialysis
T CD8 CD28−/*μ*l	64.3 ± 67.3 (43.1; 14.0–85.4)	112.1 ± 100.0 (86; 14–85.4)	**0.048**	1 w.	25 ± 33 (15; 5–30)	**0.008**	**<0.001**
				1 m.	57 ± 61 (35; 15–89)	*0.663*	**0.042**
				3 m.	127 ± 145 (61; 48–185)	*0.135*	*0.509*
				6 m.	243 ± 266 (122; 75–363)	**0.003**	*0.231*
				12 m.	277 ± 270 (216; 89–393)	**<0.001**	*0.054*

T CD8 CD28− %T	4.7 ± 4.4 (3.2; 1.2–6.0)	9.7 ± 6.0 (9.7; 5.6–15.5)	**0.004**	1 w.	3.6 ± 3.7 (2.4; 1.0–5.0)	*0.411*	**<0.001**
				1 m.	4.8 ± 5.0 (2.4; 1.0–7.9)	*0.772*	**0.004**
				3 m.	8.2 ± 6.7 (5.5; 3.7–10.5)	*0.067*	*0.409*
				6 m.	14.8 ± 11.5 (11.4; 6.0–18.5)	**<0.001**	*0.320*
				12 m.	17.8 ± 14.9 (14.7; 7.1–22.6)	**<0.001**	*0.239*
				7.9 ± 2.2 y.	9.3 ± 7.1 (7.2; 3.7–13.6)	**0.010**	*0.461*

T CD8 CD28− % T CD8	27.3 ± 15.1 (22.9; 17.1–51.0)	32.7 ± 18.4 (34.6; 17.1–51)	0.244	1 w.	32.7 ± 17.0 (32.1; 16.6–41.1)	*0.292*	*1.000*
				1 m.	34.2 ± 20.0 (29.9; 17.8–52.2)	*0.570*	*0.892*
				3 m.	48.5 ± 19.9 (51.2; 31.8–62.4)	**0.002**	**0.040**
				6 m.	57.6 ± 18.5 (56.9; 45.1–73.6)	**<0.001**	**<0.001**
				12 m.	58.2 ± 19.3 (59.3; 42.3–73.7)	**<0.001**	**<0.001**
				7.9 ± 2.2 y.	43.4 ± 21.3 (42.0; 26.9–58.3)	**0.010**	*0.123*

**Table 6:** Whole blood gene expression in kidney transplant recipients (data presented as ΔΔCT).

Gene	Control group mean ± SD (median; IQR)	Time from KTx	Mean ± SD (median; IQR)	*p*-KTx vs. control
*CD4*	0.00 ± 1.01 (0.44; -0.52–0.65)	1 week	1.27 ± 1.71 (1.04; 0.58–1.62)	**0.003**
		1 month	0.95 ± 0.98 (0.76; 0.50–0.99)	**0.010**
		3 months	1.26 ± 0.94 (1.02; 0.79–1.74)	**<0.001**
		6 months	1.28 ± 1.42 (1.10; 0.53–1.68)	**0.001**
		12 months	0.64 ± 1.24 (0.76; 0.33–1.10)	**0.028**
		7.9 ± 2.2 y.	0.31 ± 1.25 (0.56; -0.08–1.14)	*0.171*

*CD8*	0.00 ± 1.43 (0.02; -0.56–1.06)	1 week	0.47 ± 2.15 (-0.09; -0.89–1.26)	*0.949*
		1 month	0.95 ± 1.43 (0.62; -0.02–1.57)	*0.146*
		3 months	1.80 ± 1.68 (1.38; 0.64–2.64)	**0.003**
		6 months	2.49 ± 2.00 (2.05; 1.45–3.26)	**<0.001**
		12 months	1.49 ± 0.92 (1.36; 0.89–2.07)	**0.001**
		7.9 ± 2.2 y.	0.84 ± 1.57 (0.94; 0.38–1.55)	*0.090*

*CTLA4*	0.00 ± 1.07 (-0.10; -0.76–0.72)	1 week	0.80 ± 1.75 (0.37; -0.54–2.27)	*0.950*
		1 month	0.36 ± 1.27 (-0.01; -0.47–1.04)	*0.786*
		3 months	0.50 ± 1.49 (0.30; -0.41–0.83)	*0.840*
		6 months	1.18 ± 1.47 (0.85; 0.08–2.14)	**0.038**
		12 months	0.47 ± 1.38 (0.27; -0.21–0.70)	*1.000*
		7.9 ± 2.2 y.	0.03 ± 1.12 (0.02; -0.66–0.83)	*0.900*

*FOXP3*	0.00 ± 0.73 (0.15; -0.33–0.42)	1 week	0.35 ± 1.45 (0.03; -0.67–0.78)	*1.000*
		1 month	−0.20 ± 0.89 (-0.03; -0.63–0.19)	*0.730*
		3 months	0.00 ± 1.20 (-0.04; -0.60–0.45)	*0.728*
		6 months	0.27 ± 1.02 (0.19; -0.40–0.90)	*1.000*
		12 months	−0.13 ± 0.81 (-0.22; -0.54–0.05)	*1.000*
		7.9 ± 2.2 y.	−0.30 ± 0.92 (-0.39; -0.95–0.30)	*0.846*

*GZMB*	0.00 ± 1.94 (0.37; -1.28–1.56)	1 week	−0.09 ± 2.16 (-0.34; -1.38–0.83)	*0.728*
		1 month	0.70 ± 1.51 (0.80; -0.31–1.40)	*0.505*
		3 months	1.42 ± 1.79 (1.18; 0.08–2.19)	*0.200*
		6 months	2.15 ± 1.74 (1.98; 1.20–3.03)	**0.004**
		12 months	1.35 ± 1.14 (1.38; 0.75–1.98)	**0.046**
		7.9 ± 2.2 y.	0.46 ± 2.09 (0.99; -0.47–1.94)	*0.708*

*IL10*	0.00 ± 1.46 (-0.29; -1.13–0.91)	1 week	3.98 ± 2.56 (4.20; 2.11–5.51)	**<0.001**
		1 month	1.29 ± 2.16 (0.76; 0.13–2.44)	**0.045**
		3 months	1.32 ± 1.72 (1.21; -0.02–2.06)	**0.035**
		6 months	2.40 ± 2.05 (2.36; 0.91–3.88)	**<0.001**
		12 months	0.61 ± 2.09 (0.22; -1.10–2.17)	*0.305*
		7.9 ± 2.2 y.	0.90 ± 1.67 (0.92; -0.44–1.94)	*0.082*

*IL2RA*	0.00 ± 0.66 (-0.04; -0.57–0.62)	1 week	0.43 ± 1.53 (-0.03; -0.58–1.03)	*1.000*
		1 month	−0.06 ± 0.86 (-0.08; -0.66–0.34)	*1.000*
		3 months	0.07 ± 1.22 (-0.08; -0.56–0.71)	*1.000*
		6 months	0.26 ± 1.13 (0.19; -0.35–1.03)	*1.000*
		12 months	−0.34 ± 0.71 (-0.25; -0.71–0.03)	*1.000*
		7.9 ± 2.2 y.	0.02 ± 0.87 (0.08; -0.78–0.80)	*0.993*

*IL4*	0.00 ± 1.00 (-0.28; -0.77–0.78)	1 week	0.27 ± 4.18 (-1.10; -2.47–2.13)	*0.514*
		1 month	0.87 ± 2.01 (0.45; -0.57–2.15)	*0.259*
		3 months	1.29 ± 2.10 (0.90; -0.09–3.01)	*0.210*
		6 months	1.11 ± 2.34 (0.48; -0.57–2.21)	*0.372*
		12 months	1.24 ± 2.33 (0.70; -0.29–1.73)	*0.495*
		7.9 ± 2.2 y.	−0.54 ± 1.14 (-0.16; -1.45–0.31)	*0.476*

*NOTCH*	0.00 ± 0.67 (-0.06; -0.42–0.55)	1 week	0.14 ± 1.30 (-0.22; -0.45–0.15)	*1.000*
		1 month	−0.30 ± 0.72 (-0.48; -0.65 to -0.15)	**0.040**
		3 months	0.23 ± 1.05 (-0.04; -0.35–0.34)	*0.963*
		6 months	0.41 ± 1.04 (0.03; -0.29–0.96)	*1.000*
		12 months	−0.01 ± 0.63 (-0.05; -0.37–0.30)	*1.000*
		7.9 ± 2.2 y.	0.20 ± 0.92 (0.05; -0.46–0.74)	*0.860*

*PDCD1*	0.00 ± 0.92 (0.24; -0.73–0.67)	1 week	0.14 ± 2.24 (-0.43; -1.26–1.28)	*0.950*
		1 month	−0.29 ± 1.33 (-0.54; -1.00 to -0.07)	*0.225*
		3 months	0.65 ± 1.68 (0.18; -0.49–1.54)	*1.000*
		6 months	1.54 ± 1.79 (1.24; 0.40–2.67)	**0.009**
		12 months	0.35 ± 1.39 (0.13; -0.21–1.06)	*1.000*
		7.9 ± 2.2 y.	−0.04 ± 1.41 (0.27; -0.78–0.91)	*0.787*

*PRF1*	0.00 ± 1.63 (0.43; -0.91–1.28)	1 week	−0.17 ± 1.87 (-0.16; -1.38–0.53)	*0.746*
		1 month	0.24 ± 1.11 (0.28; -0.53–0.78)	*0.888*
		3 months	0.97 ± 1.43 (0.81; -0.02–1.50)	*0.468*
		6 months	1.42 ± 1.63 (1.17; 0.44–2.51)	*0.066*
		12 months	0.77 ± 1.52 (1.14; 0.43–1.73)	*0.475*
		7.9 ± 2.2 y.	0.56 ± 1.99 (0.94; -0.02–1.96)	*0.438*

*TGFB*	0.00 ± 1.38 (0.24; -0.53–0.98)	1 week	1.47 ± 1.54 (1.31; 1.00–1.62)	**0.002**
		1 month	0.97 ± 0.76 (0.91; 0.58–1.14)	**0.031**
		3 months	1.28 ± 1.11 (1.04; 0.70–1.45)	**0.010**
		6 months	1.36 ± 1.30 (1.27; 0.80–1.84)	**0.003**
		12 months	0.71 ± 1.25 (0.98; 0.53–1.38)	*0.054*
		7.9 ± 2.2 y.	0.46 ± 1.65 (0.76; 0.16–1.25)	*0.146*

*TNFA*	0.00 ± 1.17 (0.17; -0.41–0.76)	1 week	0.51 ± 1.54 (0.17; -0.58–1.55)	*0.960*
		1 month	0.51 ± 1.02 (0.48; -0.19–0.98)	*0.904*
		3 months	0.76 ± 1.22 (0.40; -0.05–1.39)	*0.755*
		6 months	0.74 ± 1.26 (0.56; -0.21–1.77)	*0.732*
		12 months	0.26 ± 0.94 (0.04; -0.35–0.73)	*0.909*
		7.9 ± 2.2 y.	0.41 ± 1.25 (0.52; -0.12–1.13)	*0.708*

*TNFRSF18*	0.00 ± 1.12 (0.10; -0.53–0.47)	1 week	0.95 ± 1.24 (0.69; 0.15–2.01)	*0.051*
		1 month	0.92 ± 0.86 (0.94; 0.58–1.21)	**0.016**
		3 months	0.92 ± 0.95 (0.89; 0.41–1.35)	**0.042**
		6 months	1.03 ± 1.40 (0.84; 0.02–1.84)	*0.052*
		12 months	0.51 ± 1.79 (1.10; 0.13–1.55)	*0.064*
		7.9 ± 2.2 y.	1.02 ± 3.54 (0.47; -0.16–1.13)	*0.124*

## Data Availability

The data used to support the findings of this study are available from the corresponding author upon request.
